# Professional Etiquette

**Published:** 1865-09

**Authors:** L. C. Ingersoll

**Affiliations:** Keokuk, Iowa


### Original Essays and Communications.

### PROFESSIONAL ETIQUETTE.

BY L. C. INGERSOLL, KEOKUK, IOWA.

[Read before the Iowa State Dental Society.]

Harmony is the oil which causes the machinery of society to run smoothly. If it freely flows up through the rock on which the social organization is built, such organization is rich indeed. No body of men, no class in society, can sustain mutual relations to each other without a most careful observance, in good faith, of certain rules to secure harmony. The tangential force of selfishness in society is strong, and drives asunder and scatters men who do not consider themselves sacredly, though voluntarily, bound by the rules generally observed in the intercourse of high minded men with each other. Those rules which come into existence in civilized society by mutual consent and general observance, are termed rules of etiquette.

It is etiquette that holds the machinery of society together. There is neighborhood etiquette, business etiquette, drawing-room etiquette, parliamentary etiquette, professional etiquette. Wherever and whenever men are drawn together, from whatever cause or occasion, be it their similar manual or professional pursuits, or the coalescing of their ideas, or the accidental circumstance of travel even, instinct and common sense prescribe etiquette.

Etiquette is often understood to consist in a set of rigid characterless rules—an arbitrary limiting of the conduct of man among men—a system of exactions unnecessarily, if not unjustly proscribing our liberties. One man goes forth with the restricted idea of being answerable for his demeanor only to the laws of the land; and standing within the limit of the statute he says: 'Outside of and beyond this I am my own master—I will do as I please—it is no bodies business how or what I do, if I do not break the laws of the Commonwealth.' Another man goes forth, and, without asking for the limit of the law, he determines to conform his conduct to the rule of right—and furthermore asks himself, 'How can I make my presence and business relations acceptable and agreeable to my fellow men, so that we may live harmoniously and pleasantly together, and be helped by others while I help them?' The *manner of the man* toward his fellow men, especially toward those of his own class, rank, or profession, is what we term *etiquette.*

Originally, the term etiquette was applied to a card of observances, written out and handed to each of the guests at grand entertainments given by persons of high rank, or a card prescribing dress, style of manners, and method of approach to a king, queen, or other dignitary. These rules were so rigidly enforced that the person disregarding, or being indifferent to them, was most unceremoniously thrust out from the presence and society he fain had sought, and stigmatized with shame and disgrace. But in our day and in our country, rules of etiquette are not so rigidly enforced. For the most part men are left to demean themselves among men according to their own sense of honor, and that respect which is due to man from man. So a man may act the gentlemen, or the boar,—the man of honor, or the knave, and society must endure it.

In the earlier days of dentistry, before dentistry had attained to the rank of a profession, every day's experience brought something new into the world, which the lucky possessor held as a dental wonder—a secret to be most jealously guarded, lest some other venturer like himself should get an inkling of it. To succeed in this he must turn his back on his neighbor dentist, and make rigid his rule of non-intercourse, lest that natural friendship, which exists between man and his fellow, should beguile him into some *lapsus linguæ*, and he should find his peculiar thunder heralding some other man's skill as wonderful as his own. These were the days of infancy. These were the days of ignorance. But now that infancy has grown to manhood, and ignorance has given place to general enlightment,—now that the practice of dentistry has became a well defined system, and has taken rank as a profession, infancy and ignorance can no longer excuse dishonorable and unprofessional practices of any sort. Dentistry, as a profession, has no secrets to guard and no jealousies to foster. Dental colleges, dental literature, dental societies and dental conventions make, what is known in dentistry, public property, available to all who desire to know. This advance step places dentists on a new platform, and makes the difference among dentists to consist not in the number and variety of the secrets they claim to be possessed of, but in their capacities to comprehend the nature of their calling, and in their skill to accomplish. These are natural differences, and will not, among honorable men, ever be made a barrier to harmony and professional intercourse. But there are certain bad elements in human nature—selfishness, injustice and meanness—which, unrestrained, will break the harmony of any society, on whatever basis formed. Now self interest and selfishness are widely different—the one honorable and right, the other dishonorable and horribly wicked. *Self-interest* leads a man to look to his own interest *because it is his own*, when he can do so without hazarding the interests of another, which are just as sacred as his own. Selfishness, on the other hand, looks after ones own interests, regardless of the interests of others, to the hazard, or even ruin, of other men's interests. Selfishness *may* accept the dictates of honor and justice, but only at convenience. Let a man go into a profession with these bad tendencies of human nature unrestrained and his profession will be made only a show-case for exhibiting them.

Society expects of him that he will act worthly his part—that from the higher level of his profession he will be an example of noble, manly bearing, working harmoniously with others who occupy a like position for their united welfare, and for the welfare of mankind.

An honorable high minded man needs not rules of etiquette prescribed for him, more than an honest man needs legislative enactments against theft. He falls readily and naturally into a course of conduct, which so commends itself to all honorable men that they feel like reciprocating it. This brings professional harmony. Let dentists every where take the stand of honor, and rules of etiquette will lie filed away unobserved yet well observed.

Let us ever bear in mind that the standing of the dental profession, in the community and among other professions, is what our standing is as men, that it is not the profession that makes the man, but the man the profession. If it were within the power of this society to execute, we should resolve, first, to make men, and secondly, to make dentists—not first a dentist, then a man.

A high standard of pressional etiquette is ethical; in other words, it involves moral character. A man who is base and mean professionally, is base and mean at heart. He is void of moral principle. The man who, with jealous eye, will seek, or take, an opportunity offered to injure the reputation of another in the profession, who is honorably and honestly pursuing it; or will use underhanded or unwarrantable means to injure his business and build up his own, has actually an element of baseness in his character, and is unworthy of the confidence of the community or the respect of the profession. From the seeds of moral principles such noxious weeds do not grow. "Do men gather grapes of thorns or figs of thistles?" To secure a high standard of professional etiquette, characterless, base, low-minded men should be discouraged, and if possible prevented, from entering the profession, and only those encouraged who possess high toned moral instincts as a basis of an honorable professional bearing.

With such material and on such a basis we can fraternize. Fraternity is the deity we have invoked in the organization of the profession of Iowa. Fraternity has been the tutelary angle in convening us from time to time; has abode with us in our gatherings; has, at each successive meeting, linked strangers with us and made us friends together; and if we be true men, true to ourselves and true to the profession, fraternity will bring to our number every true man of the State.

